# Electrohypersensitivity: what is belief and what is known?

**DOI:** 10.3389/fpubh.2025.1603692

**Published:** 2025-05-19

**Authors:** Frank de Vocht, Martin Röösli

**Affiliations:** ^1^Population Health Sciences, Bristol Medical School, University of Bristol, Bristol, United Kingdom; ^2^NIHR Applied Research Collaboration West (ARC West), Bristol, United Kingdom; ^3^Swiss TPH (Swiss Tropical and Public Health Institute), Allschwil, Switzerland; ^4^University of Basel, Basel, Switzerland

**Keywords:** electrohypersensitivity, electromagnetic radiation, electromagnetic fields, IEI-EMF, provocation (challenge) tests, research methodologies and methods

## Abstract

Electrohypersensitivity (EHS), or idiopathic environmental intolerance attributed to electromagnetic fields (IEI-EMF), is a condition with fluid and transient symptoms associated to exposure to non-ionizing radiation by people claiming to suffer from it. However, the scientific evidence linking the exposure to the reported effects to datwe has eluded researchers, and may not even exist. In the current perspective we outline what is objectively known about EMF as the cause for EHS and what is based on anecdotal information only. We discuss why randomized provocation trials were considered the most appropriate research methodology to elucidate causal links between EMF exposure and effects in a scientifically robust manner, what the main arguments against such studies are, and whether these criticisms are valid. Finally, we synthesize the evidence and beliefs around EHS and provide future directions of research and practice.

*“The human understanding, when it has once adopted an opinion, draws all things else to support and agree with it.” (Francis Bacon, Novum Organum, Book I, Aphorism 46).*

Electrohypersensitivity (EHS), also known by other names including ‘microwave syndrome’, ‘microwave illness’, ‘electromagnetic hypersensitivity’, ‘electrosensitivity’, ‘hypersensitivity to electricity’, or ‘Wifi allergy’, has been known for decades, but has become virulent in the 1980s, when office workers, especially from Scandinavia, reported health problems related to video display unit (VDU) work (1–4). While at that time neurasthenic and skin symptoms were reported most often, subsequently, over time the most commonly reported symptoms evolved into headaches, sleep disturbances, concentration difficulties, fatigue and tinnitus, among many others (5–12).

Based on self-reported population surveys, the prevalence of EHS was found to vary between countries and years, including for example 1.5% in Sweden in 1997 (13) and 2.6–3.2% about a decade later (2), 3.2% in California in 1998 (14), 2.0 and 3.5% in Austria in 1994 and 2008 (15), 3.5% in the Netherlands in 2011 (16), 5% in Switzerland in 2004 (17), about 10% in Germany in 2004 (18), 13% in Taiwan in 2007 (19) and 4% in Taiwan five years later. There is no clear pattern with EMF exposure levels, with repeated surveys indicating the population prevalence to have stabilized since the early 2000s despite changing exposure patterns and sources (20) (although prevalence data for the last decade are scarce). Repeated surveys in the same individuals indicated that self-reported EHS is a relatively transient statement (21, 22). Cluster analyses have also not identified any specific symptom clusters that are related to specific EMF exposure sources or to EMF exposure in general (7). The pattern of symptoms is not part of any recognized syndrome (23).

Due to similarities to other forms of idiopathic environmental intolerance (IEI), such as multiple chemical sensitivity, EHS is more accurately referred to as IEI attributed to EMF (IEI-EMF) (24, 25). For comparison, the population prevalences for IEI-EMF are lower than self-reported sensitivity (IEI) to noise, odor and chemicals (26).

A substantial part of afflicted individuals report to react to EMF exposure within minutes (7, 9, 27). The obvious way to study acute effect is thus through randomized controlled trials. In these so called provocation studies participants usually undergo a series of randomly assigned exposure and control conditions (crossover design). A recent review of 40 such human provocation studies concluded that under blinded conditions people suffering from IEI-EMF cannot identify exposures better than by chance nor were their symptom experiences different from control populations, or different from the general population (11, 12). There has been criticism of these provocation trials, which, in addition to standard methodological quality issues such as appropriate blinding, boil down to the following points (12, 28, 29):

- *Provocation studies only capture short term effects on self-perceived health*. This is correct, but triangulates with what a substantial proportion of EHS people report. Using a transdisciplinary approach, it therefore makes sense that the academic community has focussed on the complaints of the afflicted individuals. Using experimental approaches to study effects of long-term exposure is usually not possible due to ethical and logistic reasons. However, observational research on non-specific symptoms and long-term radiofrequency EMF exposure also did not find indications for an association, although with lower certainty of evidence (30).
- *The effect of EMF is diluted or masked because there are too many participants enrolled in these studies who are not “true EHS” or only experience EHS to some lesser degree.* This claim has been discussed by Bosch-Capblanch et al. (11), who highlight that this has been tested directly through the application of individualized tests (in which no susceptible individual was identified) (31–34) and through provocation studies with so-called open provocations (32, 35–38), where study participants were informed about the true exposure status. These studies unequivocally demonstrated that study participants reported symptoms if they were aware of the EMF exposure status, but no EMF effect was observed by the same participants under blinded conditions (11). In some experiments (39, 40) study participants even reported symptoms if they were falsely told they were exposed to EMF while in truth they were not. There is also substantial evidence showing that experimentally induced expectations of harmful EMF leads to elevated symptom reports (40–42). Altogether these findings demonstrate that for IEI-EMF psychogenic reactions play an important role.
- *Provocation studies used a single exposure specification with respect to frequency and intensity, while people with EHS claim to each respond to different characteristics of the EMF exposure*. Indeed most studies applied only one to two exposure conditions. However, some studies addressed this concern using an individualized exposure set-up, where exposure specifications were determined together with the study participants. But these studies did also not identify sensitive individuals (11, 32). Further, contemporary provocation studies (11, 32, 35–38) included non-blinded, so-called open, assessments to determine the individual exposure characteristics participants were sensitive to, prior to the blinded provocation trials.
- *Provocation trials cause too much pressure to succeed for participants.* It has been argued that this distress may mask any effects from the EMF exposure. Some pressure to succeed for study participants is comprehensible. For this reason, contemporary provocation studies have been conducted with pre-trial acclimatization sessions or even conducted all trials in participants’ homes (11). There was no indication that such a study provided different results (32).
- *Provocation studies test the wrong biological mechanism.* For example, it has been proposed that repeated low dose exposures to EMF of sensitized individuals leads to enhanced responses which, it has been argued, operates through excessive oxidative stress (43). There is no empirical evidence that convincingly proves this hypothesis, nor does this triangulate well with the reported pattern of symptom development reported by IEI-EMF individuals and on which the idea of using provocation studies was based. Nonetheless, there may be some value in further investigating such protracted mechanisms.
- *People with “real” EHS will not participate in such provocation studies because they do not trust the medical establishment to do such studies well.* In the absence of any method of distinguishing “real” from “false” EHS, there is no way of solving this conundrum. In principle such studies could, of course, also be conducted by patient groups as long as they adhere to scientific standards.

The above refuting of the main criticisms of EHS provocation trials is not done to dismiss IEI-EMF may be related to EMF exposure in favor of a psychogenic explanation. Rather, we outlined these points above to emphasize that, at present, there is no robust empirical evidence that favors EMF as the causal agent (29, 44). Indeed, although through the scientific method it is not possible to prove that a single EHS individual does not exist, it has also not proven that such an individual does exist. This does not automatically imply that differences in susceptibility to EMF do not exist. As with many pathologies, differences in sensitivity to causative factors may well exist for exposure to EMF as well. For instance, research has demonstrated a broad variability, but not hypersensitivity, in the population when it comes to the perception of static electric fields (45–47) or to electric current perception thresholds (48). For hypersensitivity to biological agents, most notably various allergies, the biological mechanism is well understood. For many environmental agents such as noise or chemicals, the most established pathway for hypersensitivity is psychological via perception processing (49).

There are case reports of people who reported health complaints related to EMF exposure [for example (50)]: and indeed sometimes it is claimed without being aware that they have been exposed to EMF. These are sometimes considered as proof for a causal association (51). In contrast, there are also case studies concluding IEI-EMF was related to psychological stress (52). It is well known that anecdotes can be very convincing, and that often their impact trumps that of more robust evidence; quantitative or other. However, it has also been well established that anecdotes and case studies are extremely susceptible to various forms of bias and should not be used to infer causality. Generally, there is always a selection behind which anecdotes are put forward, or which cases are reported on, and which relies on reported effects and assumed association with the exposure above any other considerations. Thus, anecdotes and case studies can be good starting point for further experiment or other study, but cannot by themselves serve as causal proof (51, 53, 54).

Another interesting perspective put forward is that vulnerability in itself cannot be directly perceived by people. For example, people cannot know whether they are sensitive to ionizing radiation or whether they will get lung cancer after years long smoking, both established risk factors. It has thus been proposed that one should study correlations between EMF exposure and acute or long-term effects on biomarkers such as oxidative stress, hormones or protein alterations (29, 55). It would thus be valuable to conduct such studies to try and identify any biological markers of EMF-susceptibility independent of self-reports. Many studies have addressed objective biomarkers and could not identify any association with EMF below regulatory limits (56–60). If such research is conducted explicitly in the frame of IEI-EMF, a number of problems arise: The search for such biomarkers suffers from circular reasoning in that to test biomarkers one needs to identify people with EHS for comparison with people without EHS, which in the absence of biomarkers can only be identified through self-reports; the initial problem we were trying to solve. An alternative strategy describes an agnostic approach of screening a large group of people for a plethora of biological responses following exposure (or at different levels of EMF exposure) and explore whether some biological responses are more frequent in some individuals or differ between exposure conditions. Such studies have been conducted and some of these found indications that EHS individuals have higher sympathetic activity such as higher pulse rates, higher skin conductance (61). However, these studies, by design, are not able to distinguish whether the difference is caused by physical exposure or by distress, since IEI-EMF individuals feel continuously threated by EMF in their daily life. Moreover, this approach may identify individual variability in biological responses to EMF, but says little about adverse health effects. If such approaches are used to discriminate between true and false EHS, they would additionally require study population sizes orders of magnitude larger than any studies conducted to date to determine any biological signal with some scientific confidence. Obviously, specificity and sensitivity would be low, unless a very novel marker is identified. A common biological marker would also inevitably fall foul of the criticisms of the provocation trials highlighted above.

And finally, it has been put forward that EHS has been acknowledged by some authorities, e.g., by paying disability compensations (28). However, to the best of our knowledge, such decision is not evaluating disease causation but rather takes into account the functional impairment of IEI-EMF and the impact it can have on individuals’ life (62).

Considering the above, there are a several observations relevant going forward. Provocation studies have convincingly demonstrated that EMF exposure cannot be felt by people with EHS better than chance or the general population, and that EMF exposure is not causally related to short-term effects of relevant magnitude exceeding background variability from, for example, stress. In the absence of a causal link between IEI-EMF and EMF various alternative explanations for the development of IEI-EMF has been proposed (63, 64): the *sensory processing hypothesis* assumes a general higher sensitivity of sensory processing independent of EMF exposure. The *cognitive hypothesis* assumes that occurrence of symptoms results from the belief in EMF harmfulness, promoting nocebo responses to perceived EMF exposure. According to the *attributive hypothesis,* individuals suffering from pre-existing conditions search for an explanation and discover EMF as a potential cause resulting in being convinced to be exposed to EMF. Recent work indicates that the latter may be the most relevant (65).

In terms of IEI-EMF symptoms and EMF exposure, there is little value in continuing the same research. It would therefore be prudent to consider other provocation designs, for example the single-case repeated design (66), above labeled as individualized tests. Alternatively, we propose an exposure-disease model that relies on long-term exposure rather than relatively short high exposures and focusses on the whole population rather than on self-perceived hypersensitivity.

Nevertheless, such research does not mean that we should not care about people feeling affected by EMF exposure. In fact, despite observed EHS prevalences in the range of a few percent, only a small minority is severely suffering in their daily life. For instance, in a three-year environmental counseling study in the German part of Switzerland only 70 individuals per year asked for medical advice despite advertising the study to relevant stakeholder groups (67). Recently, in Switzerland a consultation project for EHS individuals was initiated (https://www.mednis.ch/de). In the first year of operation (autumn 2023 to 2024) about 60 patients asked for a specialized consultation. For the treatment of EHS individuals, no guidelines have been developed so far. From the above mentioned Swiss counseling project (67) it was concluded that focus should be on the treatment of the disease independent of the patient’s causal model. For some patients who are anxious owed to their daily EMF exposed, cognitive behavioral therapy has been beneficial (68). In practice it may be prudent for society to try and make amendments, within reason, where family, friends, colleagues or staff identify as suffering from EHS; regardless of whether the underlying mechanism is biophysical or psychological in nature.

## Data Availability

The original contributions presented in the study are included in the article/supplementary material, further inquiries can be directed to the corresponding author/s.

## References

[1] Berg M Axelson O. Evaluation of a questionnaire for facial skin complaints related to work at visual display units (1990) 22:71–7. doi: 10.1111/j.1600-0536.1990.tb01520.x, PMID: 2323206

[2] Hagstrom M Auranen J Johansson O Ekman R. Reducing electromagnetic irradiation and fields alleviates experienced health hazards of VDU work. Pathophysiology. (2012) 19:81–7. doi: 10.1016/j.pathophys.2012.01.005, PMID: 22364840

[3] Liden S Berg M. Skin problems in users of video display terminals. Discrepancy Between Subjective Symptoms Objective Signs 3. (1991) 156:18. 1828647

[4] Swanbeck G Bleeker T. Skin problems from visual display units. Provocation Skin Symptoms Under Exp Cond. (1989) 69:46. 2563608

[5] Hillert L Hedman BK Soderman E Arnetz BB. Hypersensitivity to electricity: working definition and additional characterization of the syndrome. J Psychosom Res. (1999) 47:429–38. doi: 10.1016/S0022-3999(99)00048-3, PMID: 10624841

[6] Oftedal G Wilen J Sandstrom M Mild KH. Symptoms experienced in connection with mobile phone use. Occup Med Lond. (2000) 50:237–45. doi: 10.1093/occmed/50.4.237, PMID: 10912374

[7] Röösli M Moser M Baldinini Y Meier M Braun-Fahrländer C. Symptoms of ill health ascribed to electromagnetic field exposure - a questionnaire survey. Int J Hyg Environ Health. (2004) 207:141–50. doi: 10.1078/1438-4639-00269, PMID: 15031956

[8] Eltiti S Wallace D Zougkou K Russo R Joseph S Rasor P. Development and evaluation of the electromagnetic hypersensitivity questionnaire. Bioelectromagnetics. (2007) 28:137–51. doi: 10.1002/bem.20279, PMID: 17013888

[9] Baliatsas C Van Kamp I Bolte J Schipper M Yzermans J Lebret E. Non-specific physical symptoms and electromagnetic field exposure in the general population: can we get more specific? Systematic Review Environ Int. (2012) 41:15–28. doi: 10.1016/j.envint.2011.12.002, PMID: 22245541

[10] Andrianome S De Seze R Braun A Selmaoui B. Descriptive self-reporting survey of people with idiopathic environmental intolerance attributed to electromagnetic fields (IEI-EMF): similarities and comparisons with previous studies. J Public Health-Heid. (2018) 26:461–73. doi: 10.1007/s10389-017-0886-0

[11] Bosch-Capblanch X Esu E Oringanje CM Dongus S Jalilian H Eyers J. The effects of radiofrequency electromagnetic fields exposure on human self-reported symptoms: a systematic review of human experimental studies. Environ Int. (2024) 187:108612. doi: 10.1016/j.envint.2024.108612, PMID: 38640611

[12] Schmiedchen K Driessen S Oftedal G. Methodological limitations in experimental studies on symptom development in individuals with idiopathic environmental intolerance attributed to electromagnetic fields (IEI-EMF) - a systematic review. Environ Health. (2019) 18:88. doi: 10.1186/s12940-019-0519-x, PMID: 31640707 PMC6805477

[13] Hillert L Berglind N Arnetz BB Bellander T. Prevalence of self-reported hypersensitivity to electric or magnetic fields in a population-based questionnaire survey. Scand J Work Environ Health. (2002) 28:33–41. doi: 10.5271/sjweh.644, PMID: 11871850

[14] Levallois P Neutra R Lee G Hristova L. Study of self-reported hypersensitivity to electromagnetic fields in California. Environ Health Perspect. (2002) 110:619–23. doi: 10.1289/ehp.02110s4619, PMID: 12194896 PMC1241215

[15] Schröttner J Leitgeb N. Sensitivity to electricity – temporal changes in Austria. BMC Public Health. (2008) 8:310. doi: 10.1186/1471-2458-8-310, PMID: 18789137 PMC2562386

[16] Baliatsas C Bolte J Yzermans J Kelfkens G Hooiveld M Lebret E. Actual and perceived exposure to electromagnetic fields and non-specific physical symptoms: an epidemiological study based on self-reported data and electronic medical records. Int J Hyg Environ Health. (2015) 218:331–44. doi: 10.1016/j.ijheh.2015.02.001, PMID: 25704188

[17] Schreier N Huss A Röösli M. The prevalence of symptoms attributed to electromagnetic field exposure: a cross-sectional representative survey in Switzerland. Sozial-und Praventivmedizin. (2006) 51:202–9. doi: 10.1007/s00038-006-5061-2, PMID: 17193782

[18] Blettner M Schlehofer B Breckenkamp J Kowall B Schmiedel S Reis U. Mobile phone base stations and adverse health effects: phase 1 of a population-based, cross-sectional study in Germany. Occup Environ Med. (2009) 66:118–23. doi: 10.1136/oem.2007.037721, PMID: 19017702

[19] Meg Tseng MC Lin YP Cheng TJ. Prevalence and psychiatric comorbidity of self-reported electromagnetic field sensitivity in Taiwan: a population-based study. J Formos Med Assoc. (2011) 110:634–41. doi: 10.1016/j.jfma.2011.08.005, PMID: 21982467

[20] Huang PC Cheng MT Guo HR. Representative survey on idiopathic environmental intolerance attributed to electromagnetic fields in Taiwan and comparison with the international literature. Environ Health. (2018) 17:5. doi: 10.1186/s12940-018-0351-8, PMID: 29334987 PMC5769530

[21] Traini E Martens AL Slottje P Vermeulen RCH Huss A. Time course of health complaints attributed to RF-EMF exposure and predictors of electromagnetic hypersensitivity over 10 years in a prospective cohort of Dutch adults. Sci Total Environ. (2023) 856:159240. doi: 10.1016/j.scitotenv.2022.159240, PMID: 36209879

[22] Röösli M Mohler E Frei P. Sense and sensibility in the context of radiofrequency electromagnetic field exposure. Comptes-Rendus Physique de l’Académie des Sciences. (2010) 11:576–84. doi: 10.1016/j.crhy.2010.10.007

[23] ANSES. Hypersensibilité électromagnétique ou intolérance environnementale idiopathique attribuée aux champs électromagnétiques. Avis de l’Anses. Rapport d’expertise collective. Agence nationale de sécurité sanitaire de l’alimentation, de l’environnement et du travail. (2018). Available at: https://www.anses.fr/en/system/files/AP2011SA0150Ra.pdf

[24] WHO. Fact sheet 296: Electromagnetic fields and public health - Electromagnetic Hypersensitivity. Available online at: https://www.who.int/peh-emf/publications/facts/fs296/en/.2005.

[25] Rubin GJ Nieto-Hernandez R Wessely S. Idiopathic environmental intolerance attributed to electromagnetic fields (formerly 'electromagnetic hypersensitivity'): an updated systematic review of provocation studies. Bioelectromagnetics. (2010) 31:1–11. doi: 10.1002/bem.20536, PMID: 19681059

[26] Nordin S Karvala K Nyback MH Sainio M. Prevalence of environmental annoyance in a Swedish and Finnish general population: impact of everyday exposures on affect and behavior. J Environ Psychol. (2018) 56:84–90. doi: 10.1016/j.jenvp.2018.03.004

[27] Baliatsas C Van Kamp I Lebret E Rubin GJ. Idiopathic environmental intolerance attributed to electromagnetic fields (IEI-EMF): a systematic review of identifying criteria. BMC Public Health. (2012) 12:643. doi: 10.1186/1471-2458-12-643, PMID: 22883305 PMC3504528

[28] Bevington M. The effects of radiofrequency electromagnetic fields exposure on human self-reported symptoms: A systematic review of human experimental studies. Environ Int. (2024) 187:108888:108612. doi: 10.1016/j.envint.2024.108888 38640611

[29] Leszczynski D. Review of the scientific evidence on the individual sensitivity to electromagnetic fields (EHS). Rev Environ Health. (2022) 37:423–50. doi: 10.1515/reveh-2021-0038, PMID: 34229366

[30] Röösli M Dongus S Jalilian H Eyers J Esu E Oringanje CM. The effects of radiofrequency electromagnetic fields exposure on tinnitus, migraine and non-specific symptoms in the general and working population: a systematic review and meta-analysis on human observational studies. Environ Int. (2024) 183:108338. doi: 10.1016/j.envint.2023.108338, PMID: 38104437

[31] Radon K Maschke C. Gibt es Elektrosensibilität im D-Netzbereich? Umweltmed Forsch Prax. (1998) 3:125.

[32] van Moorselaar I Slottje P Heller P van Strien R Kromhout H Murbach M. Effects of personalised exposure on self-rated electromagnetic hypersensitivity and sensibility - a double-blind randomised controlled trial. Environ Int. (2017) 99:255–62. doi: 10.1016/j.envint.2016.11.031, PMID: 27939951

[33] Verrender A Loughran SP Dalecki A Freudenstein F Croft RJ. Can explicit suggestions about the harmfulness of EMF exposure exacerbate a nocebo response in healthy controls? Environ Res. (2018) 166:409–17. doi: 10.1016/j.envres.2018.06.032, PMID: 29936289

[34] Verrender A Loughran SP Dalecki A McKenzie R Croft RJ. Pulse modulated radiofrequency exposure influences cognitive performance. Int J Radiat Biol. (2016) 92:603–10. doi: 10.1080/09553002.2016.1213454, PMID: 27501119

[35] Eltiti S Wallace D Ridgewell A Zougkou K Russo R Sepulveda F. Does short-term exposure to mobile phone base station signals increase symptoms in individuals who report sensitivity to electromagnetic fields? A double-blind randomized provocation study. Environ Health Perspect. (2007) 115:1603–8. doi: 10.1289/ehp.10286, PMID: 18007992 PMC2072835

[36] Verrender A Loughran SP Anderson V Hillert L Rubin GJ Oftedal G. IEI-EMF provocation case studies: a novel approach to testing sensitive individuals. Bioelectromagnetics. (2018) 39:132–43. doi: 10.1002/bem.22095, PMID: 29125197

[37] Wallace D Eltiti S Ridgewell A Garner K Russo R Sepulveda F. Do TETRA (airwave) base station signals have a short-term impact on health and well-being? A randomized double-blind provocation study. Environ Health Perspect. (2010) 118:735–41. doi: 10.1289/ehp.0901416, PMID: 20075020 PMC2898847

[38] Wallace D Eltiti S Ridgewell A Garner K Russo R Sepulveda F. Cognitive and physiological responses in humans exposed to a TETRA base station signal in relation to perceived electromagnetic hypersensitivity. Bioelectromagnetics. (2012) 33:23–39. doi: 10.1002/bem.20681, PMID: 21647932

[39] Szemerszky R Koteles F Lihi R Bardos G. Polluted places or polluted minds? An experimental sham-exposure study on background psychological factors of symptom formation in 'Idiophatic environmental intolerance attributed to electromagnetic fields'. Int J Hyg Environ Health. (2010) 213:387–94. doi: 10.1016/j.ijheh.2010.05.001 20538519

[40] Brascher AK Schulz SM Van den Bergh O Witthoft M. Prospective study of nocebo effects related to symptoms of idiopathic environmental intolerance attributed to electromagnetic fields (IEI-EMF). Environ Res. (2020) 190:110019. doi: 10.1016/j.envres.2020.110019, PMID: 32777274

[41] Brascher AK Raymaekers K van den Bergh O Witthoft M. Are media reports able to cause somatic symptoms attributed to WiFi radiation? An experimental test of the negative expectation hypothesis. Environ Res. (2017) 156:265–71. doi: 10.1016/j.envres.2017.03.040, PMID: 28371755

[42] Witthoft M Rubin GJ. Are media warnings about the adverse health effects of modern life self-fulfilling? An experimental study on idiopathic environmental intolerance attributed to electromagnetic fields (IEI-EMF). J Psychosom Res. (2013) 74:206–12. doi: 10.1016/j.jpsychores.2012.12.002, PMID: 23438710

[43] Stein Y Udasin IG. Electromagnetic hypersensitivity (EHS, microwave syndrome) - review of mechanisms. Environ Res. (2020) 186:109445. doi: 10.1016/j.envres.2020.109445, PMID: 32289567

[44] de Vocht F. The influence of Maslow's hammer. Response to: electromagnetic hypersensitivity close to mobile phone base stations - a case study in Stockholm, Sweden. Rev Environ Health. (2023) 38:753–4. doi: 10.1515/reveh-2022-0058, PMID: 35447024

[45] Jankowiak K Driessen S Kaifie A Kimpeler S Krampert T Kraus T. Identification of environmental and experimental factors influencing human perception of DC and AC electric fields. Bioelectromagnetics. (2021) 42:341–56. doi: 10.1002/bem.22347, PMID: 33973657

[46] Kursawe M Stunder D Krampert T Kaifie A Driessen S Kraus T. Human detection thresholds of DC, AC, and hybrid electric fields: a double-blind study. Environ Health. (2021) 20:92. doi: 10.1186/s12940-021-00781-4, PMID: 34419058 PMC8380375

[47] Kursawe M Kaifie A Krabbe J Kimpeler S Kuhn R Kraus T. The role of the DC component in human perception of AC-DC hybrid electric fields and a comparison with the AC component. Sci Rep. (2023) 13:16320. doi: 10.1038/s41598-023-43556-2, PMID: 37770510 PMC10539523

[48] Schrottner J Leitgeb N Hillert L. Investigation of electric current perception thresholds of different EHS groups. Bioelectromagnetics. (2006) 28:208–13. doi: 10.1002/bem.20294, PMID: 17080457

[49] Nordin S Neely G Olsson D Sandstrom M. Odor and noise intolerance in persons with self-reported electromagnetic hypersensitivity. Int J Environ Res Public Health. (2014) 11:8794–805. doi: 10.3390/ijerph110908794, PMID: 25166918 PMC4198991

[50] Hardell L Nilsson M. Summary of seven Swedish case reports on the microwave syndrome associated with 5G radiofrequency radiation. Rev Environ Health. (2024) 40:147–57. doi: 10.1515/reveh-2024-0017, PMID: 38889394

[51] Moore EM Johnson SB. Research and statistics: case reports, anecdotal evidence, and descriptive epidemiologic studies in pediatric practice. Pediatr Rev. (2009) 30:323–4. doi: 10.1542/pir.30-8-323 19648264

[52] Domotor Z Szabolcs Z Berdi M Witthoft M Koteles F Szemerszky R. An idiographic approach to idiopathic environmental intolerance attributed to electromagnetic fields (IEI-EMF) part I. Environmental, psychosocial and clinical assessment of three individuals with severe IEI-EMF. Heliyon. (2022) 8:e09987. doi: 10.1016/j.heliyon.2022.e09987, PMID: 35874058 PMC9305360

[53] Fagerlin A Wang C Ubel PA. Reducing the influence of anecdotal reasoning on people's health care decisions: is a picture worth a thousand statistics? Med Decis Mak. (2005) 25:398–405. doi: 10.1177/0272989X05278931, PMID: 16061891

[54] Shermer M. How anecdotal evidence can undermine scientific results. Sci Am. (2008). Available at: https://www.scientificamerican.com/article/how-anecdotal-evidence-can-undermine-scientific-results/

[55] Belpomme D Carlo GL Irigaray P Carpenter DO Hardell L Kundi M. The critical importance of molecular biomarkers and imaging in the study of Electrohypersensitivity. A scientific consensus international report. Int J Mol Sci. (2021) 22:7321. doi: 10.3390/ijms22147321, PMID: 34298941 PMC8304862

[56] Pw Kenny R Evelynne Johnson E Adesanya AM Richmond C Beyer F Calderon C. The effects of radiofrequency exposure on male fertility: a systematic review of human observational studies with dose-response meta-analysis. Environ Int. (2024) 190:108817. doi: 10.1016/j.envint.2024.108817, PMID: 38880061

[57] Johnson EE Kenny RPW Adesanya AM Richmond C Beyer F Calderon C. The effects of radiofrequency exposure on adverse female reproductive outcomes: a systematic review of human observational studies with dose-response meta-analysis. Environ Int. (2024) 190:108816. doi: 10.1016/j.envint.2024.108816, PMID: 38880062

[58] Benke G Abramson MJ Brzozek C McDonald S Kelsall H Sanagou M. The effects of radiofrequency exposure on cognition: a systematic review and meta-analysis of human observational studies. Environ Int. (2024) 188:108779. doi: 10.1016/j.envint.2024.108779 38821015

[59] Meyer F Bitsch A Forman HJ Fragoulis A Ghezzi P Henschenmacher B. The effects of radiofrequency electromagnetic field exposure on biomarkers of oxidative stress in vivo and in vitro: a systematic review of experimental studies. Environ Int. (2024) 194:108940. doi: 10.1016/j.envint.2024.108940, PMID: 39566441

[60] Pophof B Kuhne J Schmid G Weiser E Dorn H Henschenmacher B. The effect of exposure to radiofrequency electromagnetic fields on cognitive performance in human experimental studies: systematic review and meta-analyses. Environ Int. (2024) 191:108899. doi: 10.1016/j.envint.2024.108899, PMID: 39265322

[61] Hug K Röösli M. Elektromagnetische Hypersensitivität - Bewertung von wissenschaftlichen Studien. Stand Ende 2011, Umwelt-Wissen Nr. 1218. Bundesamt für Umwelt: Bern. (2012).

[62] Bosch-Capblanch X Esu E Moses Oringanje C Dongus S Jalilian H Eyers J. Response to letter from Bevington M., Electrosensitivity UK. Environ Int. (2024) 191:108982. doi: 10.1016/j.envint.2024.108982, PMID: 39213920

[63] Dieudonne M. Electromagnetic hypersensitivity: a critical review of explanatory hypotheses. Environ Health. (2020) 19:48. doi: 10.1186/s12940-020-00602-0, PMID: 32375774 PMC7201940

[64] Bordarie J Ledent M Dieudonne M Choisay F De Clercq E. Could electrohypersensitivity be a specific form of high sensory processing sensitivity? Front Public Health. (2025) 13:1550427. doi: 10.3389/fpubh.2025.1550427, PMID: 40093733 PMC11906343

[65] Ariccio S Traini E Portengen L Martens A Slottje P Vermeulen R. Chicken or egg? Attribution hypothesis and nocebo hypothesis to explain somatization associated to perceived RF-EMF exposure. Front Public Health. (2025) 13:13. doi: 10.3389/fpubh.2025.1561373, PMID: 40270737 PMC12014461

[66] Miller MG Corner J. The 'n = 1′ randomized controlled trial. Palliat Med. (1999) 13:255–9. doi: 10.1191/026921699674890886, PMID: 10474715

[67] Röösli M Frei P Bolliger-Salzmann H Barth J Hlavica M Huss A. Umweltmedizinisches Beratungsnetzwerk von Hausärzten: ein Schweizer Pilotprojekt. Umweltmedizin in Forschung und Praxis. (2011) 16. Available at: http://edoc.unibas.ch/entities/publication/5a9e43e1-5fe7-42b1-bd90-cd1718db3f18

[68] Rubin GJ Das Munshi J Wessely S. A systematic review of treatments for electromagnetic hypersensitivity. Psychother Psychosom. (2006) 75:12–8. doi: 10.1159/000089222, PMID: 16361870

